# Multisensory Integration in the Virtual Hand Illusion with Active Movement

**DOI:** 10.1155/2016/8163098

**Published:** 2016-10-25

**Authors:** Woong Choi, Liang Li, Satoru Satoh, Kozaburo Hachimura

**Affiliations:** ^1^Advanced Production Systems Engineering Course, National Institute of Technology, Gunma College, Maebashi, Japan; ^2^College of Information Science and Engineering, Ritsumeikan University, Kusatsu, Japan; ^3^Gungin System Service Co., Ltd., Maebashi, Japan

## Abstract

Improving the sense of immersion is one of the core issues in virtual reality. Perceptual illusions of ownership can be perceived over a virtual body in a multisensory virtual reality environment. Rubber Hand and Virtual Hand Illusions showed that body ownership can be manipulated by applying suitable visual and tactile stimulation. In this study, we investigate the effects of multisensory integration in the Virtual Hand Illusion with active movement. A virtual xylophone playing system which can interactively provide synchronous visual, tactile, and auditory stimulation was constructed. We conducted two experiments regarding different movement conditions and different sensory stimulations. Our results demonstrate that multisensory integration with free active movement can improve the sense of immersion in virtual reality.

## 1. Introduction

The mutual interaction of the sensory signals is a critical aspect in human perception and cognition. Recently, with the development of virtual reality (VR) technology, increasing researches were carried out on projecting multisensory information to virtual representations of the actual body. Ideally, the virtual representations in VR space should be identical to the actual body. However, in practice, physical differences arise due to the spatial limitation when constructing the VR space. The real-time representation of multisensory information plays a pivotal role in an immersive VR environment.

The shifts between physical stimuli and human perceptions are known as illusions. Using these sensory distortions, more realistic perceptions can be represented despite the physical limitations of sensory display interfaces.

Body ownership illusion, which is typically induced by VR, has been widely studied in the past few decades. Self-recognition is necessary for human cognition adapting to changes in the environment [1]. Mental representation of one's own body, which is called body image, is not limited to the sense of body ownership but also comprises the multisensory perceptions such as visual and tactile information [2]. In addition, body image can be extended to an object or an artificial limb attached to the human body. Therefore, body image can be intentionally manipulated by displaying coherent multisensory information.

A famous illusion of body ownership is the Rubber Hand Illusion (RHI) [3–7]. In the RHI, the subjects viewing stimulation of a rubber hand being stroked synchronously with their own unseen hand feel that the rubber hand is part of their own body [8].

The displacement of body ownership has also been observed in VR environment, in which a virtual hand was displayed as the visual stimulation [9, 10]. This illusion is called the Virtual Hand Illusion (VHI). As reported by IJsselsteijn et al., the VR environment produced a weaker illusion than the RHI but a more convincing subjective illusion than the mixed reality environment [11]. Furthermore, connectivity of the virtual hand with the rest of the virtual body has a significant effect on the subjective illusion of ownership [12].

It is relatively easy to evoke the illusory ownership by displaying synchronous visual and tactile stimulation with multisensory display interface. Therefore, with a proper integration and display of multisensory stimulation, a more realistic experience can be elicited in an immersive virtual reality environment.

Many researchers investigated the effect of synchrony for visual, tactile, and auditory stimulation. The results showed that the synchronous conditions led to stronger illusions of body ownership than the asynchronous conditions [4, 12–14]. In other words, synchronous stimulation is critical for inducing the VHI. Most studies on synchrony of multisensory stimulation are focused on examining the minimum conditions necessary to induce the illusion of body ownership. However, considering that the synchronous stimulation is the basis of multisensory integration, we aimed to investigate the most effective combination for inducing the illusion of ownership over the virtual hand in a 3D virtual reality environment.

In practice, a human being moves his/her body parts initiatively and receives subsequent multisensory feedback. Therefore, investigation of active movement in the VHI is necessary for answering the question of how to properly extend body image in VR space. Recent research showed that the change of body image is affected not only by passive sensory stimulation but also by the feedback from active movement. When visual and felt movements were synchronized, active movement arises stronger illusion than passive movement [15]. It has confirmed that illusory body ownership existed during active virtual games in an immersive VR space [16]. There is also evidence that the VHI can be induced by only synchronous visual and motor stimulation, in the absence of tactile stimulation [14].

In VR environment, active movement and multisensory feedback produce the sense of ownership and the sense of agency [17]. Most researches on the VHI in active movement using VR representation were focused on the visual and tactile stimulation. It was reported that the inclusion of a sound cue heightened the effects of the illusion and caused participants to more readily accept the rubber hand into the body schema [18]. Under the invisible hand illusion, the influence of visual-tactile integration on proprioceptive updating is modifiable by irrelevant auditory cues merely through the temporal correspondence between the visual-tactile and auditory events [19]. However, the effects of visual, tactile, and auditory integration in the VHI have not been studied yet.

The purpose of our study was to investigate the effects of multisensory integration in the VHI with active movement. In this paper, we constructed a VR system that interactively generates synchronous visual, tactile, and auditory stimulation. Our system enables participants to perform active movement in a VR environment. We conducted two experiments: (1) the VHI in different active movement conditions and (2) multisensory integration in the VHI. The effect of the visual presentation of the virtual hand was also evaluated.

## 2. Materials and Methods

### 2.1. Participants

Twenty participants (19 males) with mean age 19.0 ± 1.83 (SD) were recruited for the experiments. All participants had normal or corrected-to-normal vision and were right-handed. None had previously participated in similar studies.

All of the participants gave written informed consents prior to their participation. The protocol was approved by the ethics committees of Gunma National College of Technology.

### 2.2. Experimental Setup

**Figure 1:**
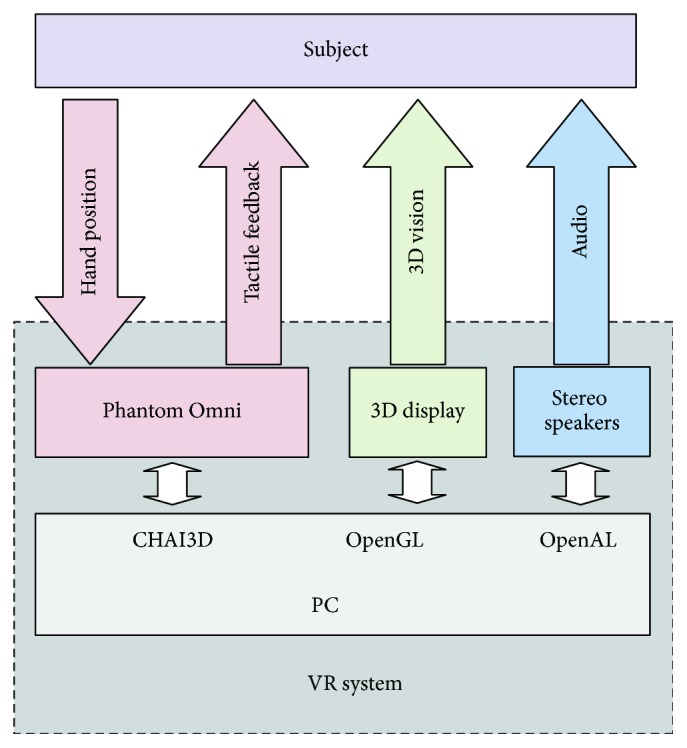
Configuration of the virtual reality system.

The virtual reality setup consisted of a stereoscopic three-dimensional (3D) display (LG FLATRON W2363D), a six degree-of-freedom haptic device (SensAble Technologies Phantom Omni), and a pair of stereo speakers (Figure 1). The display had a resolution of 1,920 × 1,080 and synchronized with active shutter 3D glasses (NVIDIA 3D Vision) at 120 Hz. The haptic device provided force feedback and positional sensing in a 160*W* × 120*H* × 70*D* mm workspace with a 0.88 N continuous exertable force and a 3.3 N maximum exertable force at nominal position. A purpose-built frame with the display mounted on its top was constructed. The haptic device and the speakers were hidden inside the frame with a curtain during the experiments.

**Figure 2:**
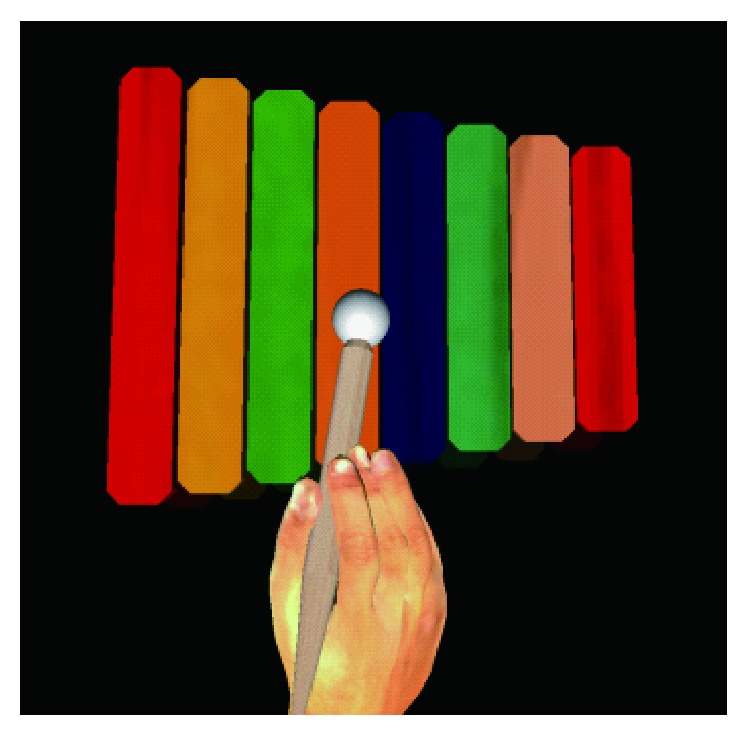
Virtual xylophone, mallet, and the virtual hand rendered and displayed in the experiments.

A virtual xylophone system was designed for this study. The system allowed active movement and interactively provided synchronous visual, tactile, and auditory stimulation in real time. In a xylophone playing task, participants saw a horizontally placed 3D virtual xylophone with a mallet (Figure 2). A 3D virtual right hand matched to their own hand sizes was also displayed in certain experimental conditions. Participants could operate the virtual hand and play the xylophone with their own unseen hand by holding and moving a pen-shaped tool of the haptic device. A tactile feedback with the resilience of a rubber-headed mallet was received when participants virtually struck the xylophone. A synchronous sound was played by the stereo speakers. The pitch and the volume of the sound were determined according to the struck bar and the striking speed. Libraries of CHAI3D, OpenGL, and OpenAL were used to build the system.

### 2.3. Procedure

Two experiments were performed in a quiet and dimly lit laboratory room.

#### 2.3.1. Experiment  1: The VHI in Different Active Movement Conditions

**Figure 3:**
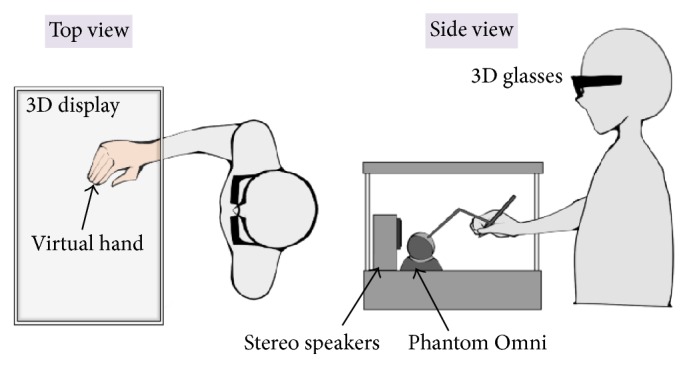
Experimental setup in experiment 1.

This experiment was aimed to investigate the VHI in different active movement conditions. Participants were seated in front of the system with their right sleeve rolled up. They were directed to correctly hold the pen-shaped tool of the haptic device with their right hand while the device remained unseen. They were then asked to look at the display through 3D glasses. The virtual hand (without the xylophone and the mallet) was displayed as the visual stimulation (Figure 3). Participants were asked to perform left/right, forward/backward, up/down, rotatory, and free movements. The duration for each condition was 30 seconds. The order of the five conditions was counter-balanced across participants.

After the experiment, participants filled in a 11-item questionnaire in Japanese as shown below.

*Questionnaire for Experiment  1*

(Q-a1) Sometimes it seemed as if my hand were located where I saw the virtual hand.
(Q-a2) Sometimes I felt as if the virtual hand were my hand.
(Q-a3) Sometimes I felt as if my hand were made by computer graphics.
(Q-a4) At some moments, it seemed as if the virtual hand began to resemble my own hand.
(Q-a5) Sometimes it seemed as if I might have more than one right hand.
(Q-a6) Sometimes I felt as if my hand were existed in the virtual environment.
(Q-a7) I had the sensation in questions (Q-a1) to (Q-a6) during left/right movements.
(Q-a8) I had the sensation in questions (Q-a1) to (Q-a6) during forward/backward movements.
(Q-a9) I had the sensation in questions (Q-a1) to (Q-a6) during up/down movements.
(Q-a10) I had the sensation in questions (Q-a1) to (Q-a6) during rotatory movements.
(Q-a11) I had the sensation in questions (Q-a1) to (Q-a6) during free movements.

Each question was scored on a 7-point Likert scale, with 1 indicating strongly disagree and 7 strongly agree. Questions (Q-a1) to (Q-a6) were partially adapted and modified from [3]. Questions (Q-a7) to (Q-a11) were introduced to apply to different conditions. For questions (Q-a7) to (Q-a11), reasons for their choice were asked.

Measurement of the displacement of the perceived hand position (proprioceptive drift) is a major way to evaluate the strength of the feeling of ownership in the RHI [20, 21]. However, most of these studies were carried out in passive movement conditions, in which the participant was asked to rest his/her hand on a table. The perceived hand positions were recorded before and after the participant's hand was tapped or stroked passively by the experimenter.

The task in our study was an active movement one, which was designed to resemble a typical dynamic operation in VR environment. Measurement of the proprioceptive drift in the traditional RHI studies is difficult for us because the active hand is constantly moving. Therefore, we used the questionnaire to evaluate the strength of the illusory ownership.

#### 2.3.2. Experiment  2: Multisensory Integration in the VHI

**Figure 4:**
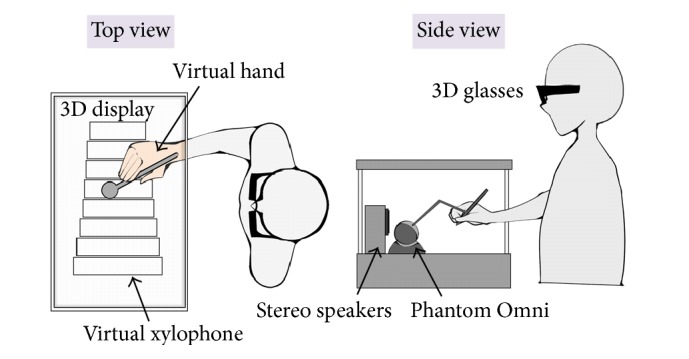
Experimental setup in experiment 2 (conditions C1, C3, C5, and C7).

This experiment was aimed at investigating the effect of multisensory integration in the VHI with active movement. The experimental setup was similar to that in experiment 1. In this experiment, eight different experimental conditions with different combinations of visual, tactile, and auditory stimulation were designed (Table 1). It should be noted that, for visual stimulation, the xylophone and the mallet were displayed in all conditions, whereas the virtual hand was only displayed in conditions C1, C3, C5, and C7. Visual, tactile, and auditory stimulation were synchronized in all conditions. The order of the eight conditions was counter-balanced across participants.  Figure 4 illustrated the experimental setup of conditions C1, C3, C5, and C7 as an example.

Participants were directed to move their hand to the reference point to begin the experiment of each condition. They were asked to strike each bar of the virtual xylophone once in an ascending scale. They were then asked to play the xylophone freely for 15 seconds. After the experiment of each condition, participants filled in a 5-item, 7-point Likert scaled questionnaire in Japanese as shown below.

*Questionnaire for Experiments  2: Common Questions*

(Q-b1) It seemed as if I were playing the xylophone with the mallet.
(Q-b2) It seemed as if I were holding the mallet.
(Q-b3) I could move the mallet to any position at my will.
(Q-b4) I felt as if I were striking an object in the virtual environment.
(Q-b5) I felt an increasing virtual reality experience during the experiment.

For conditions C1, C3, C5, and C7, five more questions that referred to the virtual hand were included as shown below.

*Questionnaire for Experiments  2: Questions for Conditions C1, C3, C5, and C7*

(Q-c1) Sometimes I felt as if the virtual hand were my hand.
(Q-c2) I felt as if my real hand were located at the virtual hand.
(Q-c3) Sometimes I felt as if my hand were made by computer graphics.
(Q-c4) At some moments, it seemed as if the virtual hand began to resemble my own hand.
(Q-c5) Sometimes it seemed as if I might have more than one right hand.

For conditions C3, C4, C7, and C8, one more question that referred to auditory stimulation was included. For conditions C5–C8, one more question that referred to tactile stimulation was included as shown below.

*Questionnaire for Experiments  2: Questions for Conditions C3–C8*

(Q-d1) It seemed as if the sound were coming from the xylophone bars where I struck.
(Q-d2) It seemed as if the force were coming from the xylophone bars where I struck.

## 3. Results

**Figure 5:**
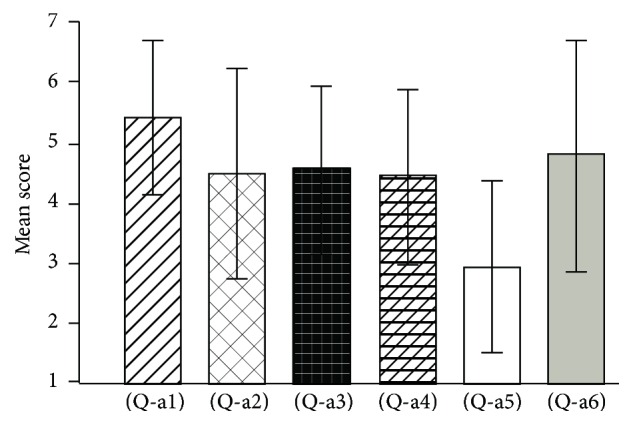
Questionnaire results for (Q-a1) to (Q-a6) in experiment 1.

**Figure 6:**
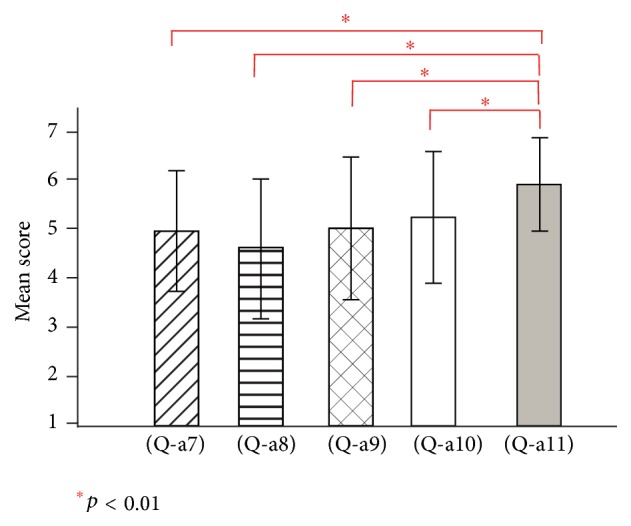
Questionnaire results for (Q-a7) to (Q-a11) in experiment 1.

Figure 5 shows the mean scores and standard deviations regarding questions (Q-a1) to (Q-a6) in experiment 1.  Figure 6, regarding questions (Q-a7) to (Q-a11), shows that free movement condition (Q-a11) has a significantly higher mean score than other conditions (paired *t*-test, *t*(19) = 3.57, *t*(19) = 3.44, *t*(19) = 3.21, *t*(19) = 2.94, and *p* < 0.01).

**Figure 7:**
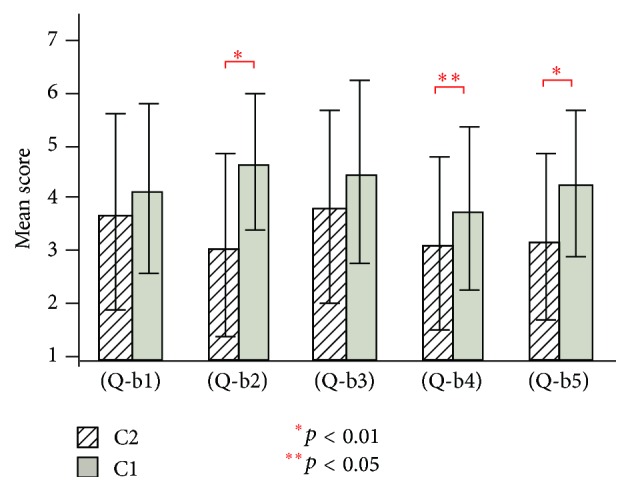
Questionnaire results of (Q-b1) to (Q-b5) for conditions C1 and C2 in experiment 2.

Figure 7 shows the results of questions (Q-b1) to (Q-b5) for conditions C1 and C2 (visual stimulation only) in experiment 2. For conditions C1 and C2, the existence of the virtual hand showed a significant effect in (Q-b2), (Q-b5) (*t*(19) = 4.29, *t*(19) = 3.57, and *p* < 0.01), and (Q-b4) (*t*(19) = 2.10, and *p* < 0.05).

**Figure 8:**
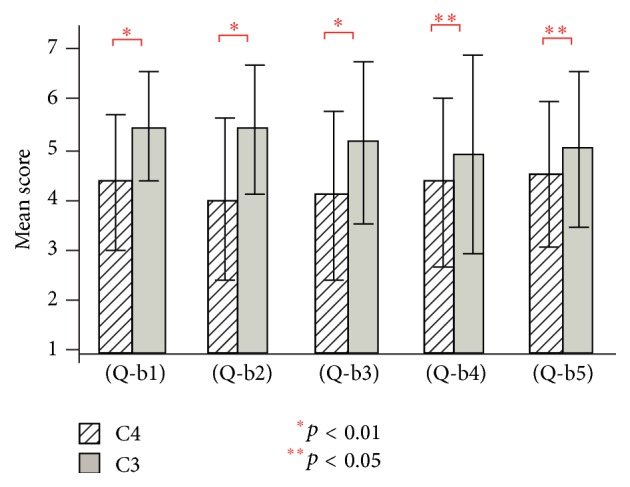
Questionnaire results of (Q-b1) to (Q-b5) for conditions C3 and C4 in experiment 2.

Figure 8 shows the results of questions (Q-b1) to (Q-b5) for conditions C3 and C4 (visual and auditory stimulation) in experiment 2. For conditions C3 and C4, the existence of the virtual hand showed a significant effect in (Q-b1), (Q-b2), and (Q-b3) (*t*(19) = 5.08, *t*(19) = 3.34, *t*(19) = 3.12, and *p* < 0.01) and (Q-b4) and (Q-b5) (*t*(19) = 2.34, *t*(19) = 2.24, and *p* < 0.05).

**Figure 9:**
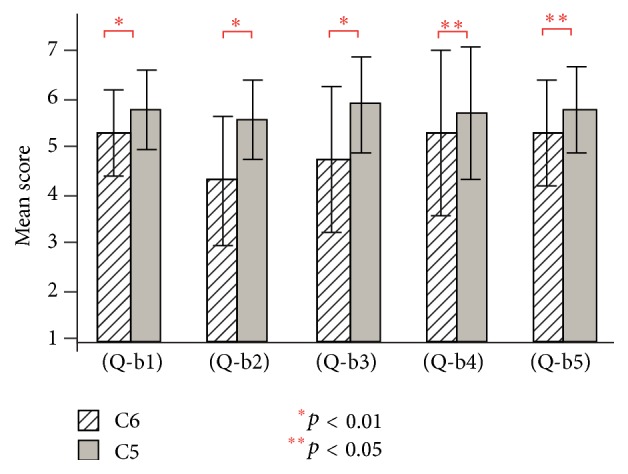
Questionnaire results of (Q-b1) to (Q-b5) for conditions C5 and C6 in experiment 2.

Figure 9 shows the results of questions (Q-b1) to (Q-b5) for conditions C5 and C6 (visual and tactile stimulation) in experiment 2. For conditions C5 and C6, the existence of the virtual hand showed a significant effect in (Q-b1), (Q-b2), and (Q-b3) (*t*(19) = 2.93, *t*(19) = 4.08, *t*(19) = 3.29, and *p* < 0.01) and (Q-b4) and (Q-b5) (*t*(19) = 2.18, *t*(19) = 2.65, and *p* < 0.05).

**Figure 10:**
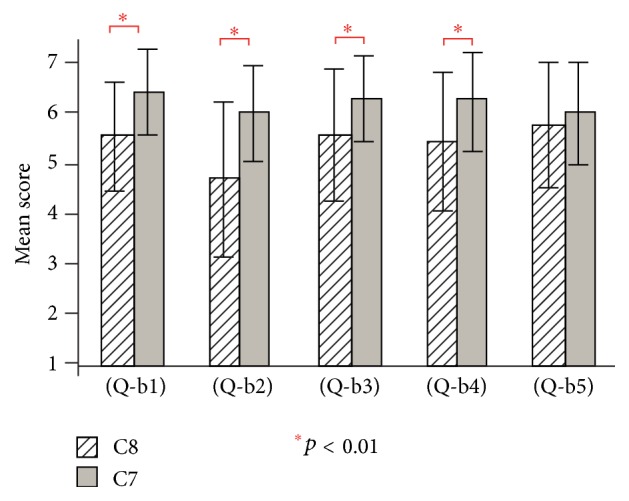
Questionnaire results of (Q-b1) to (Q-b5) for conditions C7 and C8 in experiment 2.

Figure 10 shows the results of questions (Q-b1) to (Q-b5) for conditions C7 and C8 (visual, auditory, and tactile stimulation) in experiment 2. For conditions C7 and C8, the existence of the virtual hand showed a significant effect in (Q-b1), (Q-b2), (Q-b3), and (Q-b4) (*t*(19) = 5.10, *t*(19) = 3.65, *t*(19) = 3.47, *t*(19) = 3.39, and *p* < 0.01).

**Figure 11:**
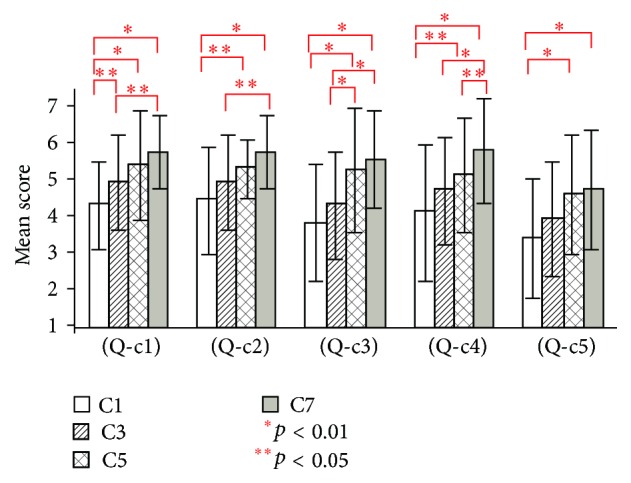
Questionnaire results of questions (Q-c1) to (Q-c5) for conditions C1, C3, C5, and C7 in experiment 2.

Figure 11 shows the results of questions (Q-c1) to (Q-c5) for conditions C1, C3, C5, and C7 (with the virtual hand) in experiment 2.

For question (Q-c1), there were significant differences between conditions C1 and C5 (*t*(19) = 3.17, *p* < 0.01), conditions C1 and C7 (*t*(19) = 5.25, *p* < 0.01), conditions C1 and C3 (*t*(19) = 2.56, *p* < 0.05), and conditions C3 and C7 (*t*(19) = 2.63, *p* < 0.05).

For question (Q-c2), there were significant differences between conditions C1 and C7 (*t*(19) = 3.27, *p* < 0.01), conditions C1 and C5 (*t*(19) = 2.67, *p* < 0.05), and conditions C3 and C7 (*t*(19) = 2.38, *p* < 0.05).

For question (Q-c3), there were significant differences between conditions C1 and C5 (*t*(19) = 4.92, *p* < 0.01), conditions C1 and C7 (*t*(19) = 6.05, *p* < 0.01), conditions C3 and C5 (*t*(19) = 2.97, *p* < 0.01), and conditions C3 and C7 (*t*(19) = 4.08, *p* < 0.01).

For question (Q-c4), there were significant differences between conditions C1 and C7 (*t*(19) = 3.61, *p* < 0.01), conditions C3 and C7 (*t*(19) = 3.17, *p* < 0.01), conditions C1 and C5 (*t*(19) = 2.50, *p* < 0.05), and conditions C5 and C7 (*t*(19) = 2.67, *p* < 0.05).

For question (Q-c5), there were significant differences between conditions C1 and C5 (*t*(19) = 3.74, *p* < 0.01) and conditions C1 and C7 (*t*(19) = 4.35, *p* < 0.01).

**Figure 12:**
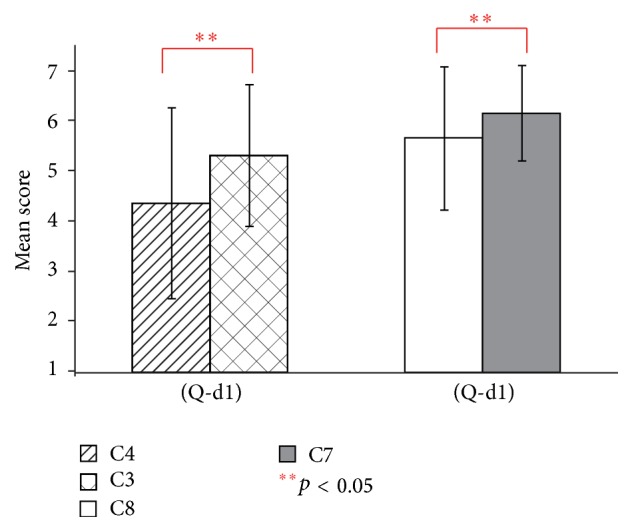
Questionnaire results of (Q-d1) in experiment 2.

**Figure 13:**
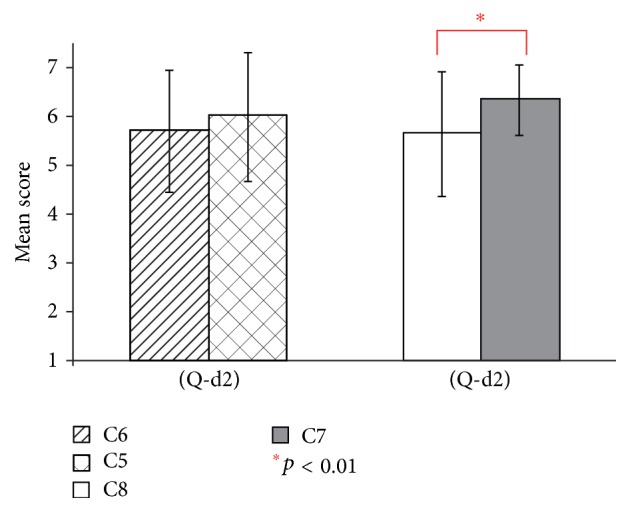
Questionnaire results of (Q-d2) in experiment 2.

Figures 12 and 13 show the result of questions (Q-d1) and (Q-d2). For (Q-d1), the conditions with the virtual hand (conditions C3 and C4) had significantly higher mean scores than those without the virtual hand (conditions C7 and C8) (*t*(19) = 2.83, *t*(19) = 2.13, and *p* < 0.05). For conditions C7 and C8, the existence of the virtual hand also showed a significant effect in (Q-d2) (*t*(19) = 3.05, *p* < 0.01). No significant difference was observed between conditions C5 and C6 in (Q-d2).

## 4. Discussions

We investigate the effects of multisensory integration in the VHI with active movement.

Experiment  1 examined the VHI in different active movement conditions.

The experimental results showed that, in translation (the movements without rotation), left/right and forward/backward movements yielded less illusion than up/down movements. Because it is relatively difficult to perceive the depth for human, the perception gap between the virtual space and the real space in vertical movements (up/down) was smaller than that in horizontal movements (left/right and forward/backward) [22, 23].

Participants reported that they had a strong illusion at the near side and felt sense of incongruity during forward/backward movements. This result that might have been caused by the difference between camera view in the virtual space and participant's view in the real space has been increased in forward/backward movements. As shown in Figure 6, questionnaire results were in agreement with that the forward/backward movement yielded least illusion.

Rotatory movements had a higher mean score than translation movements. Participants experienced stronger illusion because they felt less incongruity of spatial coordinates between virtual and real spaces especially during rotatory movements performed at the near side. Not only objective spatial parameters but also subjective impressions informed by previous experiences have an effect on the VHI [24]. Rotatory hand movements, which were less experienced than translation hand movements for participants, evoked greater illusion in our experiment.

Participants felt significantly stronger illusion in the condition of free movement. This result indicates that free-willed active movement can enhance the illusion of body ownership.

Previous studies suggested that the strongest illusion was reported when the rubber hand and the real hand were in the closest positions [25]. Furthermore, efference copy and the sensory feedback should be coincided in time for having the sense of agency [26]. Neurons in the parietal lobe related to the sense of agency function as mirror neuron [27]. It fires when an action is performed or the same action is performed by another. In the parietal lobe, the visual feedback and the predicted sensory feedback which is generated by the efference copy are compared [28]. During the free movements performed in our experiments, when the two feedbacks matched, the participants felt as if their real hand was moving in the virtual space and experienced a stronger illusion.

Experiment  2 examined the effects of multisensory integration in VHI with active movement.

We assumed that the VHI in VR space can be enhanced by applying multisensory integration to the virtual hand.

In experiment 2, conditions C1 and C2 (visual stimulation only conditions) had lower mean scores compared to the other six conditions. In addition, only three out of five items showed a significant effect of the existence of the virtual hand (Figure 7). In contrast, the existence of the virtual hand showed a significant effect in all items of the visual-auditory (Figure 8) and visual-tactile (Figure 9) conditions. It indicates that, with only the visual stimulation, it is relatively difficult to induce the illusion of body ownership despite the existence of the virtual hand. Note that, although conditions C7 and C8 (visual, tactile, and auditory conditions) had highest mean scores, the virtual hand showed less importance in one item (Figure 10). We consider that strong illusion was induced by multisensory integration even without visual existence of the virtual hand. Figure 11 showed that multisensory integration enhanced the strength of the illusory ownership. Furthermore, except for question (Q-c1), visual-auditory stimulation did not show significant advantage over visual only stimulation. However, visual-tactile stimulation showed a significantly greater effect on the illusion than visual only stimulation. This result shows that tactile signal is more critical than auditory signal in inducing the illusion of body ownership.

Figure 12 showed a significant effect of the existence of the virtual hand. It indicates that the auditory stimulation has been enhanced by the visually displayed virtual hand. The integration of visual and action auditory signals is one of the most important cues for human's spatial position perception [29].

In contrast, no significant effect of the virtual hand was observed in visual-tactile conditions (Figure 13, C5 and C6). Iriki et al. studied behavioral effects of tool-use in humans and monkeys [30, 31]. The results indicated that body representation in the brain could be changed following tool-use. Body image has been extended to the tool. Studies in VHI also reported that the illusion of body ownership can be extended to noncorporeal objects by synchronous movements [32]. Note that wooden stick shaped object without movement was reported to be not capable of inducing the illusion [21]. In our experiments, participants extended their body image to the virtual mallet instead of the virtual hand by performing active movements with synchronous visual-tactile stimulation. The strength of the illusory ownership has not reduced because the effect of the virtual hand has been substituted by the virtual mallet.

We conclude that not only visual stimulation but also multisensory integration with active movement is important to induce a strong illusion of body ownership in VR space. Furthermore, a stronger sense of immersion can be expected by performing a free movement task before the operation in VR space.

## 5. Conclusion

In this study, we constructed a VR system that provided interactive feedback of visual, tactile, and auditory stimulation. We investigated the effects of different hand moving conditions and multisensory integration in the illusion of body ownership with active movement. We designed a virtual xylophone playing task for the VHI experiments. The VR system provided synchronous visual, tactile, and auditory stimulation when the participants played the xylophone in VR environment. Furthermore, we evaluated the effect of the visual existence of the virtual hand under different sensory stimulation conditions.

The experiments showed that (1) free movement yielded strongest illusion in different active movement conditions, (2) tactile stimulation had more significant influences than auditory stimulation on the VHI, and (3) multisensory integration of visual, tactile, and auditory signals induced strongest illusion. We conclude that free active movement with multisensory feedback is the most effective way to induce the illusory ownership in VR space. This study suggested a possibility to improve the sense of immersion in VR space, provide multisensory feedback, and perform a set of free active movements before the formal operation.

We also expect that our study can improve the sense of immersion in VR based clinical applications, such as treatment for phantom limb pain [33] and pain relief during acupuncture [34]. A network of multisensory and homeostatic brain areas was reported to be responsible for maintaining a “body-matrix” [35]. We consider that, by using multisensory integration in VR space, training with virtual limb can be an effective therapeutic method for phantom limb pain experienced by amputees. The VR system used in this study can be extended to virtual rehabilitation training for patients' recovery after stroke. For further study, experiments for investigating the effectiveness of different multisensory synchrony conditions will be carried out. The changes of electromyography (EMG) [36] and position of the arm/hand [37] will be measured and analyzed quantitatively. The spatial information of the arm/hand during active movement can be obtained by using a motion capture system, which we used to develop a gesture based VR system [38].

## Figures and Tables

**Table 1:** The eight experimental conditions.

Conditions	Visual stimulation: the xylophone and the mallet	Visual stimulation: the virtual hand	Auditory stimulation	Tactile stimulation
C1	Yes	Yes	No	No
C2	Yes	No	No	No
C3	Yes	Yes	Yes	No
C4	Yes	No	Yes	No
C5	Yes	Yes	No	Yes
C6	Yes	No	No	Yes
C7	Yes	Yes	Yes	Yes
C8	Yes	No	Yes	Yes
